# Supplementary material for: Limited options for low-global-warming-potential refrigerants
Source: Nat Commun. 2017 Feb 17;8:14476. doi: 10.1038/ncomms14476 (PMC5321723; doi:10.1038/ncomms14476)
Supplement: Supplementary Information — Supplementary Table and Supplementary References [file ncomms14476-s1.pdf]

Supplementary Table 1. Full list of candidates from PubChem screening with GWP_{100} < 1000 and 300 K < T_{crit} < 420 K and results of simulation in the ideal vapor compression cycle. Listed are the PubChem compound identification number, chemical name and structure, ASHRAE refrigerant designation (where applicable), GWP_{100} (estimated by the method of Kazakov *et al.*[1] unless noted), estimated or literature value of critical temperature, coefficient of performance and volumetric capacity in the ideal vapor compression cycle with temperatures representative of an air-conditioning application, and comments denoting reason for including or deleting compound from further consideration. (A notation of "toxic" or "unstable" indicates that the fluid was dropped based on published data.) The fluids are grouped by chemical class and, within classes, listed in order of increasing critical temperature.

PubChem Number	IUPAC Name	Structure	ASHRAE Designation	GWP_{100}	T_{crit}/K	COP	Q_{vol}/ $MJ \cdot m^{-3}$	Comments
**Hydrocarbons**								
6324	ethane	CH_3-CH_3	R-170	6*	305.3†	‡		transcritical, blend comp.
6326	ethyne (acetylene)	CH≡CH		19	320.3	6.03	11.26	unstable, drop
8252	propene (propylene)	CH_2=CH-CH_3	R-1270	2*	364.2†	7.77	4.60	retain
6334	propane	CH_3-CH_2-CH_3	R-290	3*	369.9†	7.81	3.84	retain
6335	prop-1-yne	CH_3-C≡CH		2	387.4	8.29	3.05	see main text
6351	cyclopropane	c-CH_2-CH_2-CH_2-	R-C270	86	398.3†	8.30	3.33	3-mem ring, but stable
6360	2-methylpropane (isobutane)	$(CH_3)2$-CH_2-CH_3	R-600a	4	407.8†	8.13	1.59	low Q_{vol}, drop
123173	cyclopropene	c-CH_2-CH=CH-		<1	419.5	8.50	1.59	unstable, low Q_{vol}, drop
**Hydrofluorocarbons (HFCs)**								
11638	fluoromethane	CH_3F	R-41	116*	317.3†	‡		blend component, retain
6345	difluoromethane	CH_2F_2	R-32	677*	351.3†	7.68	7.29	retain
9620	fluoroethane	CH_2F-CH_3	R-161	4*	375.3†	8.07	4.12	retain
6368	1,1-difluoroethane	CHF_2-CH_3	R-152a	138*	386.4†	8.22	2.82	retain
9667	1,1,2,2-tetrafluoroethane	CHF_2-CHF_2	R-134	1120*	391.8†	8.16	2.48	blend component, retain
12584494	1-(difluoromethyl)-1,2,2,3,3-pentafluorocyclopropane	CHF_2-(-CF-CF_2-CF_2-)		156	405.5	7.77	1.05	low Q_{vol}, drop
**Hydrofluoroolefins (HFOs) and Fluoroolefins**								
8301	1,1,2,2-tetrafluoroethene	CF_2=CF_2	R-1114	6	308.4	‡		toxic, transcritical, drop
6369	1,1-difluoroethene	CF_2=CH_2	R-1132a	<1*	324.2	6.33	7.40	toxic
6339	fluoroethene	CHF=CH_2	R-1141	<1*	327.1§	6.62	8.52	retain
9665	1,1,2-trifluoroethene	CF_2=CHF	R-1123	3	343.0§	7.11	5.57	commercial interest
8302	1,1,2,3,3,3-hexafluoroprop-1-ene	CF_2=CF-CF_3	R-1216	9	359.0	7.25	2.65	toxic
2776731	2,3,3,3-tetrafluoroprop-1-ene	CH_2=CF-CF_3	R-1234yf	<1*	367.9†	7.63	2.79	commercialized
5365501	(E)-1,2-difluoroethene	CHF=CHF	R-1132(E)	1	370.5§	8.06	4.07	retain
12672	3,3,3-trifluoroprop-1-ene	CH_2=CH-CF_3	R-1243zf	<1*	376.9†	7.93	2.57	retain, but tox concerns
14310966	1,1,2-trifluoroprop-1-ene	CF_2=CF-CH_3	R-1243yc	5	379.0	7.96	2.42	CF_2 alert, drop
59951260	(E)-1,2-difluoroprop-1-ene	CHF=CF-CH_3	R-1252ye(E)	2	380.7	8.08	2.48	retain
16719786	(Z)-1,2-difluoroprop-1-ene	CHF=CF-CH_3	R-1252ye(Z)	2	380.7	8.08	2.48	retain
5708514	(Z)-1,1,1,2,4,4,4-heptafluorobut-2-ene	CF_3-CF=CH-CF_3	R-1327myz(Z)	2	381.0	7.41	1.60	low Q_{vol}, drop
69647	1,1,3,3,3-pentafluoropropene	CF_2=CH-CF_3	R-1225zc	4	381.2	7.85	1.98	low Q_{vol}, drop
5708720	(E)-1,3,3,3-tetrafluoroprop-1-ene	CHF=CH-CF_3	R-1234ze(E)	<1*	382.5†	7.94	2.24	commercialized
23237196	(E)-1,2,3,3-tetrafluoroprop-1-ene	CHF=CF-CHF_2	R-1234ye(E)	2	382.7	7.94	2.19	low Q_{vol}, drop
5708673	(Z)-1,2,3,3,3-pentafluoro-1-propene	CHF=CF-CF_3	R-1225ye(Z)	<1*	384.0§	7.85	1.91	commercial interest
521131	1,1-difluoroprop-1-ene	CF_2=CH-CH_3	R-1252zc	2	384.6	8.14	2.34	CF_2 alert, drop
22956412	1,1,3-trifluoroprop-1-ene	CF_2=CH-CH_2F	R-1243zc	1	384.9	8.09	2.25	CF_2 alert, drop
61109	1,1,3,3,3-pentafluoro-2-(trifluoromethyl)prop-1-ene	CF_2=C-$(CF_3)_2$		2	386.6	7.40	1.21	highly toxic, low Q_{vol}, drop
21731865	1,1,3,3-tetrafluoroprop-1-ene	CF_2=CH-CH_2F	R-1234zc	2	386.8	8.03	2.05	CF_2 alert, low Q_{vol}, drop
67745	1,1,2,3,3,4,4,4-octafluorobut-1-ene	CF_2=CF-CF_2-CF_3		5	389.5	7.41	1.21	CF_2 alert, low Q_{vol}, drop
5463203	(E)-1-fluoroprop-1-ene	CHF=CH-CH_3	R-1261ze(E)	1	390.7	8.27	2.52	retain
6432206	(Z)-1-fluoroprop-1-ene	CHF=CH-CH_3	R-1261ze(Z)	1	390.7	8.28	2.53	retain
6329539	(E)-1,2,3,3,3-pentafluoroprop-1-ene	CHF=CF-CF_3	R-1225ye(E)	<1*	390.8§	7.95	1.68	low Q_{vol}, drop
21096859	1,1,3,3,4,4,4-heptafluorobut-1-ene	CF_2=CH-CF_2-CF_3	R-1327czc	2	391.2	7.57	1.26	low Q_{vol}, drop

Supplementary Table 1 (continued)

PubChem Number	IUPAC Name	Structure	ASHRAE Designation	GWP_{100}	T_{crit}/K	COP	Q_{vol}/ $MJ \cdot m^{-3}$	Comments
**Hydrofluoroolefins (HFOs) and Fluoroolefins (continued)**								
3034116	(Z)-1,1,1,2,3,4,4,4-octafluorobut-2-ene	CF_3-CF=CF-CF_3	R-1318myy(Z)	2	394.5	7.50	1.09	low Q_{vol}, drop
2775851	(E)-1,1,1,2,3,4,4,4-octafluorobut-2-ene	CF_3-CF=CF-CF_3	R-1318myy(E)	5	394.5	7.60	1.11	low Q_{vol}, drop
70899	2-fluoroprop-1-ene	CH_3-CF=CH_2	R-1261	2	399.6	8.35	1.87	low Q_{vol}, drop
5462921	(Z)-1,2-difluoroethene	CHF=CHF	R-1132(Z)	1	405.8§	8.44	2.10	low Q_{vol}, drop
20496975	2-(difluoromethyl)-1,1,3,3,3-pentafluoroprop-1-ene	CF_2=C(-CHF_2)(-CF_3)		2	408.6	7.82	0.93	low Q_{vol}, drop
23237197	(Z)-1,2,3,3-tetrafluoroprop-1-ene	CHF=CF-CF_2	R-1234ye(Z)	1	409.4	8.25	1.40	low Q_{vol}, drop
12543097	(Z)-1,2,3,3,4,4,4-heptafluoro but-1-ene	CHF=CF-CF_2-CF_3	R-1327eyc(Z)	1	409.6	7.79	0.88	low Q_{vol}, drop
21096848	1,1,2,3,4,4,4-heptafluorobut-1-ene	CF_2=CF-CHF-CF_3	R-1327cye	3	411.2	7.82	0.96	low Q_{vol}, drop
12980437	2,3,3,4,4,4-hexafluorobut-1-ene	CH_2=CF-CF_2-CF_3	R-1336fyc	1	412.3	7.90	0.89	low Q_{vol}, drop
594043	1,1,2,3,3-pentafluoroprop-1-ene	CF_2=CF-CHF_2	R-1225yc	4	413.5	8.20	1.20	low Q_{vol}, drop
12576087	1,1,3,3,4,4,4-heptafluoro-2-(trifluoromethyl)but-1-ene	CF_2=C(-CF_3)CF_2-CF_3		2	415.5	7.40	0.55	low Q_{vol}, drop
12633094	(E)-1,3,3,4,4,4-hexafluorobut-1-ene	CHF=CH-CF_2-CF_3	R-1336ezc(E)	1	417.8	7.97	0.80	low Q_{vol}, drop
2774880	1,1,2,3,3,4,4-heptafluorobut-1-ene	CF_2=CF-CF_2-CHF_2	R-1327cyc	4	418.5	7.88	0.78	CF_2 alert, low Q_{vol}, drop
88954	1,1,2,3,4,4,4-heptafluoro-3-(trifluoromethyl)but-1-ene	CF_2=CF-CF(-CF_3)$_2$		4	419.3	7.42	0.54	CF_2 alert, low Q_{vol}, drop
11116025	(Z)-1,3,3,3-tetrafluoroprop-1-ene	CHF=CH-CF_3	R-1234ze(Z)	<1*	423.3†	8.42	0.92	low Q_{vol}, drop
**Hydrofluoroolefins (Cyclic)**								
23236045	1,2,3-trifluorocyclopropene	c-CHF-CF=CF-		<1	353.7	7.58	3.97	3-member ring, drop
13550690	1,2,3,3-tetrafluorocyclopropene	c-CF=CF-CF_2-	R-C1214	1	379.5	7.98	2.29	unstable, drop
12767	1,2,3,3,4,4-hexafluorocyclobutene	c-CF=CF-CF_2-CF_2-	R-C1316	1	385.7	7.69	1.54	low Q_{vol}, drop
12585099	3-(difluoromethylidene)-1,1,2,2-tetrafluorocyclopropane	CF2=(-C-CF2-CF2-)		2	395.9	7.82	1.35	low Qvol, drop
23234747	3,3-difluoro-1,2-bis(trifluoromethyl) cyclopropene	c-CF_2-C(-CF_3)=C(-CF_3)-		<1	417.3	7.70	0.60	low Q_{vol}, drop
**Olefins with Chlorine or Bromine**								
6594	1-chloro-1,2,2-trifluoroethene	CClF=CF_2	R-1113	12	380.1	8.06	2.59	CF_2 alert, drop
102335	2-chloro-1,1,3,3,3-pentafluoroprop-1-ene	CF_2=CCl-CF_3	R-1215xc	6	395.8	7.90	1.38	low Q_{vol}, drop
5463105	(E)-1-chloro-1,2-difluoroethene	CFCl-CHF	R-1112a	4	402.5	8.38	1.84	low Q_{vol}, drop
9664	2-chloro-1,1-difluoroethene	CF_2=CHCl	R-1122	4	411.0	8.44	1.61	CF_2 alert, low Q_{vol}, drop
11730	1-bromo-1,2,2-trifluoroethene	CBrF=CF_2	R-1113B1	6	411.8	8.37	1.39	CF_2 alert, low Q_{vol}, drop
13717712	(Z)-1-chloro-1,2,3,3,3-pentafluoro-prop-1-ene	CClF=CF-CF_3	R-1215yb(Z)	9	420.6	8.14	0.88	low Q_{vol}, drop
22714818	(E)-1-chloro-1,2,3,3,3-pentafluoro-prop-1-ene	CClF=CF-CF_3	R-1215yb(E)	10	420.6	8.13	0.88	low Q_{vol}, drop
**Fluorinated Alkynes**								
69654	1,1,1,4,4,4-hexafluorobut-2-yne	$CF_3C{\equiv}CCF_3$		1	320.8	4.80	3.22	low COP, drop
136491	1,2-difluoroethyne	FC≡CF		1	328.4	6.64	8.35	unstable, drop
69578	3,3,3-trifluoroprop-1-yne	CH≡C-CF_3		1	363.3	7.74	3.66	possibly stable, retain
32759	fluoroethyne	CF≡CH		1	403.7	8.41	2.43	unstable,drop
**Allenes**								
136312	1,1,3,3-tetrafluoropropa-1,2-diene	CF_2=C=CF_2		36	330.3	6.43	4.70	CF_2 alert, drop
21718125	1,1,3-trifluoropropa-1,2-diene	CF_2=C=CHF		8	362.1	7.77	3.48	CF_2 alert, drop
23235216	1,1,3,4,4,4-hexafluorobuta-1,2-diene	CF_2=C=CF-CF_3		7	388.2	7.69	1.41	CF_2 alert, low Q_{vol}, drop
10037	propa-1,2-diene	CH_2=C=CH_2		2	390.3	8.32	2.58	see main text
12636903	1,1,4,4,4-pentafluoro-3-(trifluoromethyl)buta-1,2-diene	CF_2=C=C-(CF_3)$_2$)		2	412.8	7.62	0.68	CF_2 alert, low Qvol, drop
**Ethers**								
73947	trifluoro(methoxy)methane	CF_3-O-CH_3	R-E143a	523*	377.9†	7.93	2.53	retain
10975466	trifluoro(fluoromethoxy)methane	CF_3-O-CH_2F	R-E134a	669	379.8	7.94	2.26	unstable, drop
2777154	1,1,1-trifluoro-2-(trifluoromethoxy) ethane	CF_3-CH_2-O-CF_3	R-E236fa1	679	394.1	7.72	1.23	low Q_{vol}, drop
8254	methoxymethane	CH_3-O-CH_3	R-E170	<1*	400.4†	8.31	2.79	retain

Supplementary Table 1 (continued)

PubChem Number	IUPAC Name	Structure	ASHRAE Designation	GWP_{100}	T_{crit}/K	COP	Q_{vol}/ $MJ \cdot m^{-3}$	Comments
**Ether +Alkene**								
9855459	1-(trifluoromethoxy)ethene	CF_3-O-CH=CH_2		1	314.8	4.23	4.16	low COP, drop
14474	1,1,2-trifluoro-2-(trifluoromethoxy) ethene	CF_3-O-CF=CF_2		6	358.8	7.05	2.43	CF_2 alert, drop
14617222	1,1,3,3,3-pentafluoro-2-(trifluoromethoxy)prop-1-ene	CF_3-C=CF_2-O-CF_3		2	361.7	6.63	1.77	CF_2 alert, low Q_{vol}, drop
21940994	1-fluoro-1-(trifluoromethoxy)ethene	CF_3-O-CF=CH_2		1	377.5	7.71	2.13	low Q_{vol}, drop
2783163	1,1,2,3,3-pentafluoro-3-(trifluoromethoxy)prop-1-ene	CF_2=CF-CF_2-O-CF_3		14	387.9	7.23	1.16	CF_2 alert, low Q_{vol}, drop
14875812	(E)-1,2,3,3,3-pentafluoro-1-(trifluoromethoxy)prop-1-ene	CF_3-CF=CF-O-CF_3		2	388.0	7.24	1.09	low Q_{vol}, drop
45075659	(Z)-1,2,3,3,3-pentafluoro-1-(trifluoromethoxy)prop-1-ene	CF_3-O-CF=CF-CF_3		2	388.0	7.24	1.09	low Q_{vol}, drop
82672	1,1,1,2,2-pentafluoro-2-(1,2,2-trifluoroethenoxy)ethane	CF_2=CF-O-CF_2-CF_3		4	395.9	7.35	0.69	low Q_{vol}, drop
22292402	1,1-difluoro-2,2-bis(trifluoromethoxy)ethene	CF_2=C(-O-CF_3)$_2$		1	400.1	7.28	0.64	CF_2 alert, low Q_{vol}, drop
71405026	1-[difluoro(trifluoromethoxy) methoxy]-1,2,2-trifluoroethene	CF_2=CF-O-CF_2-O-CF_3		10	401.2	7.29	0.82	low Q_{vol}, drop
**Cyclic Ethers**								
20271514	2,2,4-trifluoro-1,3-dioxole	c-CH=CF-O-CF_2-O-		1	375.7	7.83	2.08	low Q_{vol}, drop
69048191	4-(difluoromethylidene)-2,2-difluoro-1,3-dioxetane	CF_2=(-C-O-CF_2-O-)		1	377.4	7.69	1.94	CF_2 alert, low Q_{vol}, drop
12140230	2,2,3,3,4,5-hexafluorofuran	c-CF=CF-CF_2-CF_2-O-		1	388.2	7.62	1.44	low Q_{vol}, drop
69049115	2,2-difluoro-4-(fluoromethylidene)-1,3-dioxetane	CHF=(-C-O-CF_2-O-)		1	389.7	7.99	1.77	low Q_{vol}, drop
11744752	2,2,4,5-tetrafluoro-1,3-dioxole	c-CF_2-O-CF=CF-O-		1	400.0	8.03	2.38	retain
23067819	2,2,3-trifluoro-3-(1,2,2-trifluoroethenyl)oxirane	CF_2=CF-(-CF-O-CF_2-)		4	402.6	7.75	1.17	3-member ring, CF_2 alert, low Q_{vol}, drop
17798504	2-(difluoromethyl)-2,3,3-trifluorooxirane	CHF_2-(-CF-O-CF_2-)		627	408.0	8.04	1.15	low Q_{vol}, drop
**Oxygen (Ketenes)**								
136485	3,3,3-trifluoro-2-(trifluoromethyl) prop-1-en-1-one	O=C=C-(CF_3)$_2$)		1	388.0	7.58	1.38	low Q_{vol}, drop
21866237	2-fluoroethenone	O=C=CHF		1	400.7	8.41	2.37	non-fluor analog is toxic
54034777	2,3,3,4,4,4-hexafluorobut-1-en-1-one	O=C=CF-CF_2-CF_3		1	415.7	7.88	0.76	low Q_{vol}, drop
**Oxygen (Peroxides)**								
23234511	(5S)-3,3,5-trifluoro-1,2,4-trioxolane	c-CF_2-O-O-CHF-O-		537	389.7	8.03	1.79	peroxide, low Q_{vol}, drop
18386358	difluoromethylperoxy(trifluoro) methane	CF_3-O-O-CHF_2		459	397.8	7.92	1.41	peroxide, low Q_{vol}, drop
18469511	trifluoro(methylperoxy)methane	CF_3-O-O-CH_3		65	419.4	8.25	1.03	peroxide, low Q_{vol}, drop
**Hypofluorites and Hypochlorites**								
145297	fluoronitrite	FONO		77	329.1	6.74	11.08	OF, drop
25134276	[(Z)-1,2,3,3,3-pentafluoroprop-1-enyl] hypofluorite	CF_3CF=CF-OF		2	379.0	7.51	1.79	OF, low Q_{vol}, drop
17859950	difluoromethyl hypochlorite	CHF_2-O-Cl		65	380.0	8.16	3.40	OCl, drop
23379721	[difluoro(methoxy)methyl] hypofluorite	CH_3-CF_2-O-F		471	383.3	7.87	2.08	OF, low Q_{vol}, drop
20617073	2,2-difluoroethenyl hypofluorite	CF_2=CH-O-F		1	384.2	8.10	2.32	CF_2 alert, OF, drop
57828726	[fluoro(trifluoromethoxy)methyl] hypofluorite	CF_3-O-CHF-O-F		61	395.9	7.88	1.42	low Q_{vol}, drop
22084405	1,2,2,2-tetrafluoroethyl hypofluorite	CF_3-CHF-O-F		65	399.8	8.05	1.40	low Q_{vol}, drop
20089354	1,1,1,3,3,3-hexafluoropropan-2-yl hypofluorite	(CF_3)$_2$-CH-O-F		85	400.1	7.68	1.11	low Q_{vol}, drop
20600110	1,2,2-trifluoroethenyl hypofluorite	CF_2=CF-O-F		2	409.8	8.26	1.24	CF_2 alert, OF, low Q_{vol}, drop
59944631	1,1,2,3,3-pentafluoroprop-2-enyl hypofluorite	CF_2=CF-CF_2-O-F		8	413.9	7.95	0.95	CF2 alert, low Q_{vol}, drop
23576718	trifluoromethoxymethyl hypofluorite	CF_3-O-CH_2-O-F		12	415.2	8.17	1.06	low Q_{vol}, drop
18381084	fluoromethyl hypofluorite	FC-O-F		7	415.3	8.50	1.76	OF, low Q_{vol}, drop

Supplementary Table 1 (continued)

PubChem Number	IUPAC Name	Structure	ASHRAE Designation	GWP_{100}	T_{crit}/K	COP	Q_{vol}/ $MJ{\cdot}m^{-3}$	Comments
**Oxygen (Miscellaneous)**								
712	formaldehyde	CH_2=O		1	371.1	8.02	5.19	toxic, drop
3013928	2,3,3-trifluoroprop-2-enoyl fluoride	CF(=O)-CF=CF_2		11	399.7	8.09	1.56	low Q_{vol}, drop
58856309	(Z)-1,3,3,3-tetrafluoroprop-1-en-1-ol	CF_3-CH=CF-OH		1	400.9	8.02	1.34	low Q_{vol}, drop
12635489	3,3-difluoro-2-(trifluoromethyl)prop-2-enoyl fluoride	CF-C(=O)-C(=CF_2)-CF_3		5	413.4	7.89	0.89	CF_2 alert, low Q_{vol}, drop
67907	2,2,3,3,3-pentafluoropropanal	CH(=O)-CF_2-CF_3		16	413.5	8.06	0.93	low Q_{vol}, drop
12524720	1,3,3,3-tetrafluoro-2-(trifluoromethyl)prop-1-en-1-ol	OH-CF=C-$(CF_3)_2$		1	418.1	7.75	0.66	low Q_{vol}, drop
**Sulfur-containing (Thiols and Thioethers)**								
19050251	difluoromethanethiol	CHF_2-SH		1	373.0	8.07	4.01	retain
30555	trifluoromethanethiol	CF_3-SH		1	376.2	8.04	2.90	retain
136206	trifluoro(trifluoromethylsulfanyl) methane	CF_3-S-CF_3		3	377.7	7.58	2.16	low Q_{vol}, drop
4713114	1,1,2,2,2-pentafluoroethanethiol	CF_3-CF_2-SH		1	386.1	7.78	1.68	low Q_{vol}, drop
23498413	2,2,2-trifluoroethanethial	CF_3-CH=S		7	388.3	8.16	1.95	low Q_{vol}, drop
19779344	1,1,1,2,2-pentafluoro-2-(trifluoromethylsulfanyl)ethane	CF_3-S-CF_2-CF_3		3	406.2	7.58	0.86	low Q_{vol}, drop
54040926	[difluoro(trifluoromethylsulfanyl) methyl]peroxy-trifluoromethane	CF_3-O-O-CF_2-S-CF_3		5	413.4	7.39	0.66	low Q_{vol}, peroxide, drop
**Nitrogen-containing (including Amines)**								
141139	N,N,1,1-tetrafluoromethanamine	CHF_2-NF_2		20	341.6	7.16	5.24	retain
140187	N,N,N',N',N",N",N"',N"'-octafluoromethanetetramine	C-$(NF_2)_4$		898	367.6	6.75	1.45	low Q_{vol}, drop
10214404	N-(difluoromethyl)-N,1,1,1-tetrafluoromethanamine	CF_3-NF-CHF_2		49	374.6	7.57	2.07	low Q_{vol}, drop
144610	N-(difluoromethyl)-1,1,1-trifluoro-N-(trifluoromethyl)methanamine	C_3HF_8N		64	389.8	7.41	1.22	low Q_{vol}, drop
23233616	2,3,3,3-tetrafluoro-N-(trifluoromethyl)prop-1-en-1-imine	CF_3-CF=C=N-CF_3		1	390.8	7.45	1.07	low Q_{vol}, drop
13899145	2,3,4-trifluoroazete	c-N-CF=CF-CF=N-		1	397.4	8.23	1.91	low Q_{vol}, drop
9898808	2-diazo-1,1,1-trifluoroethane	N=N=CH-CF_3		70	398.4	8.18	1.54	low Q_{vol}, drop
10220269	1,2,2-trifluoro-N,N-bis(trifluoromethyl)ethenamine	CF_2=CF-N-$(CF_3)_2$		11	410.5	7.44	0.66	low Q_{vol}, drop
23235599	2,2,2-trifluoro-N-(trifluoromethyl)ethanimine	CF_3-CH=N-CF_3		54	417.1	8.01	0.83	low Q_{vol}, drop
**Inorganics**								
280	carbon dioxide	CO_2	R-744	1.00*	304.1†	‡		blend component, retain
222	ammonia	NH_3	R-717	<1*	405.4†	8.32	5.26	retain
**Current Fluids (HFCs, HCFCs, and Blends)**								
9633	pentafluoroethane	CF_3-CHF_2	R-125	3170*	339.2†	6.84	4.78	
n.a.	R-125/143a (50.0/50.0)	blend	R-507A	3985*	343.8†	7.14	4.70	
n.a.	R-32/125 (50.0/50.0)	blend	R-410A	1924*	344.5†	7.41	6.62	
n.a.	R-125/134a/143a (44.0/4.0/52.0)	blend	R-404A	3943*	345.7†	7.21	4.65	
n.a.	R-32/125/134a (23.0/25.0/52.0)	blend	R-407C	1624*	359.3†	7.70	4.69	
6372	chlorodifluoromethane	$CHClF_2$	R-22	1760*	369.3†	7.97	4.58	
13129	1,1,1,2-tetrafluoroethane	CF_3-CH_2F	R-134a	1300*	374.2†	7.96	2.99	
67940	1,1,1,2,3,3,3-heptafluoropropane	CF_3-CHF-CF_3	R-227ea	3350*	374.9†	7.51	1.88	

*Literature value from Myhre *et al.*[2] or E.U. regulation.[3]
†Literature value from the NIST REFPROP database.[4]
‡Fluid would be near-critical or supercritical in the condenser and was not simulated.
§Value estimated from normal boiling point as discussed in Methods

Supplementary References

1. Kazakov, A., McLinden, M. O., Frenkel, M. Computational design of new refrigerant fluids based on environmental, safety, and thermodynamic characteristics. *Ind. Eng. Chem. Res.* **51**, 12537-12548 (2012).

2. Myhre, G. *et al*. in Climate Change 2013: The Physical Science Basis, Fifth Assessment Report of the Intergovernmental Panel on Climate Change. (Cambridge University Press 2013).

3. European Parliament. Regulation (EU) No 517/2014 of the European Parliament and of the Council of 16 April 2014 on fluorinated greenhouse gases and repealing Regulation (EC) No 842/2006 (2014).

4. NIST Standard Reference Database 23, NIST Reference Fluid Thermodynamic and Transport Properties—REFPROP, version 9.1. (Standard Reference Data Program, National Institute of Standards and Technology, Gaithersburg, MD, 2013).
